# Tandem Thoracic Spinal Cord Lesions of Differing Pathologies: Concurrent Metastatic Lung Adenocarcinoma Lesion in Close Proximity to a Intradural Meningioma

**DOI:** 10.7759/cureus.6646

**Published:** 2020-01-13

**Authors:** Shawn S Rai, Carlos Goulart, Ziya Gokaslan, Michael Galgano

**Affiliations:** 1 Neurosurgery, State University of New York Upstate Medical University, Syracuse, USA; 2 Neurosurgery, The Warren Alpert Medical School of Brown University, Providence, USA

**Keywords:** spinal meningioma, spinal lung adenocarcinoma, coexisting spinal tumors, concurrent spinal tumors, thoracic spinal cord tumors, thoracic meningioma, thoracic metastasis

## Abstract

Simultaneously having two pathologically distinct neoplastic lesions causing critical spinal stenosis is exceedingly rare. When such lesions are near one another but occupy different spinal compartments, significant challenges arise.

We present the case of a patient with metastatic non-small cell carcinoma to the thoracic spine and an intradural meningioma occurring two spinal segments from each other. A 66-year-old female presented with one month of progressive mechanical back pain and two days of lower extremity weakness and urinary retention. She was found to have a left upper lobe lung mass. An urgent biopsy demonstrated non-small cell lung carcinoma. MRI of her thoracic spine demonstrated a T9 intradural-extramedullary enhancing lesion simultaneously with a destructive lesion of the T11 vertebral body extending into the anterior epidural space with significant cord compression at T9 and T11. The patient was taken for an urgent posterior decompression from T9 to T11, T9 left-sided pediculectomy with resection of intradural tumor, T11 corpectomy with anterior cage reconstruction, and instrumented fixation from T7 to L2. The pathology from the T9 lesion demonstrated findings consistent with a meningioma while the T11 lesion confirmed metastatic non-small cell lung adenocarcinoma. The patient improved neurologically postoperatively and regained the ability to ambulate within one week of surgery.

Pathologically distinct spinal lesions in close anatomic proximity, but in two separate compartments are exceptionally rare. We performed a simultaneous posterior approach for resection of the T9 meningioma and a T11 corpectomy for the metastatic lesion with rapid neurologic recovery.

## Introduction

Having multiple concurrent spine tumors of differing etiologies is exceedingly rare, especially in patients without neurofibromatosis or von-Hippel Lindau disease. Most cases present within the literature which contain concurrent spinal canal lesions within close proximity to one another comprise a combination of meningiomas, neurofibromas, and schwannomas [1-2]. To our knowledge, there has not been a documented case of a coexisting metastatic lesion with a meningioma. Here, we present the case of a female with two simultaneous thoracic lesions from metastatic non-small cell lung adenocarcinoma and a meningioma each resulting in critical spinal stenosis.

## Case presentation

**Figure 1:**
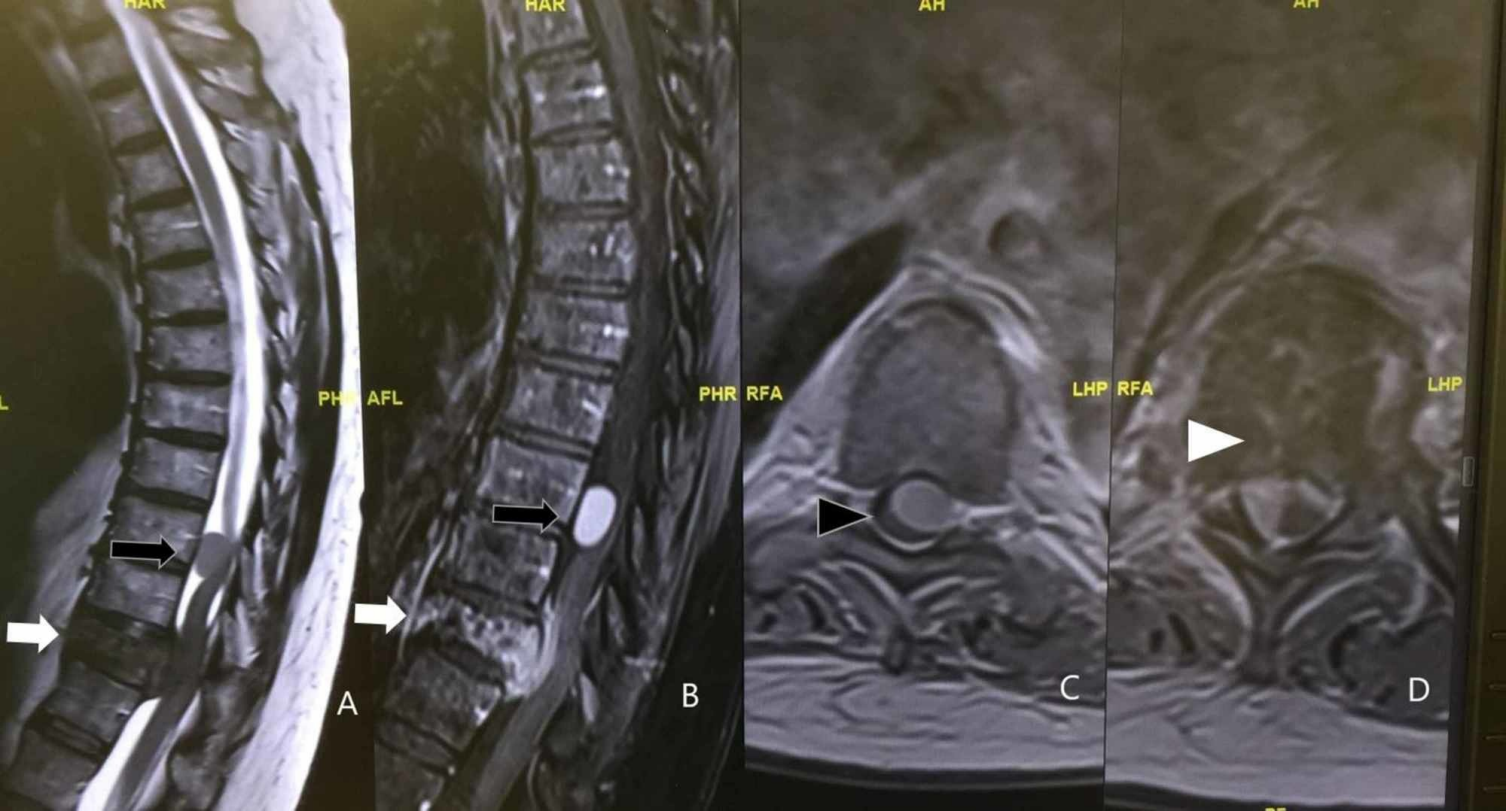
Sagittal T2-weighted without contrast (A) and sagittal T1-weighted MRI scan with gadopentetate dimeglumine contrast (B) demonstrate an intradural, extramedullary T1 enhancing lesion at T9 (black arrow) and a simultaneous destructive process of the T11 vertebral body (white arrow) with T1 enhancement of the adjacent anterior epidural space with severe spinal stenosis. Axial T1-weighted MRI scan with gadopentetate dimeglumine contrast demonstrate a intradural, extramedullary T1 enhancing lesion (black arrowhead) at the level of T9 (C) and a destructive process of the T11 vertebral body (white arrowhead) with enhancing collection on the right anterior epidural space at T11 with severe spinal stenosis (D).

**Figure 2:**
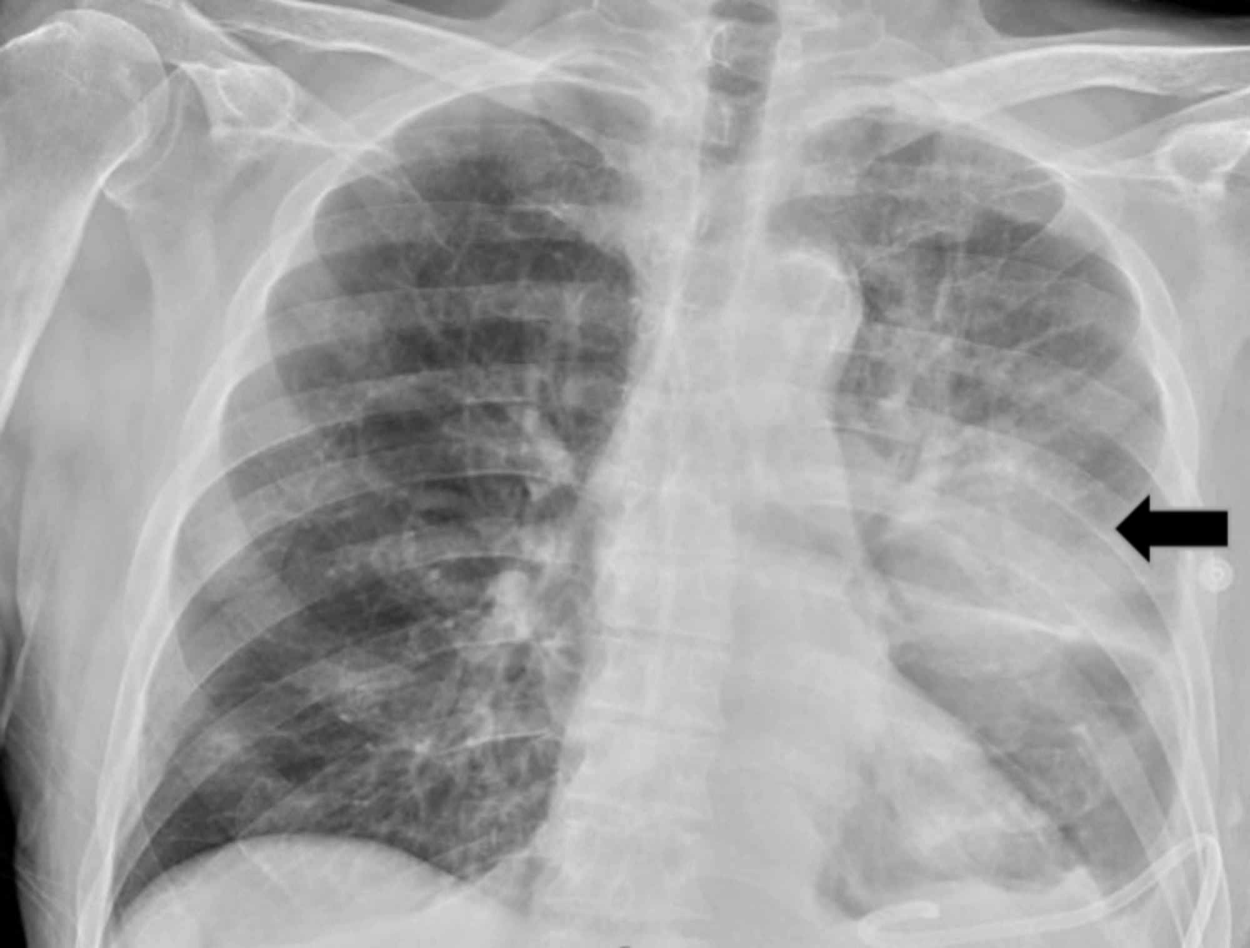
Chest radiograph demonstrating large left upper lobe lung mass (black arrow).

A 66-year-old female presented to us with a one-month history of progressive mechanical back pain and two days of subjective lower extremity weakness and urinary retention. She neurologically declined rapidly and was no longer able to ambulate upon evaluation by our service. She had no history of neurologic symptoms or deficits prior to this. MRI of her thoracic spine (Figure 1) demonstrated a T9 intradural-extramedullary enhancing lesion simultaneously with a destructive lesion of the T11 vertebral body extending into the anterior epidural space with significant cord compression at T9 and T11. She was also found to have a left upper lobe lung mass (Figure 2). An urgent lung biopsy demonstrated non-small cell lung carcinoma.

**Figure 3:**
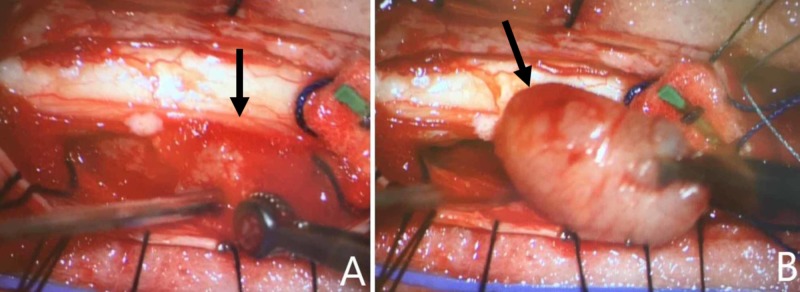
Intra-operative posterior exposure at level of T9 after completion of laminectomy and durotomy. Identification of T9 mass (A) with removal of encapsulated mass (B).

**Figure 4:**
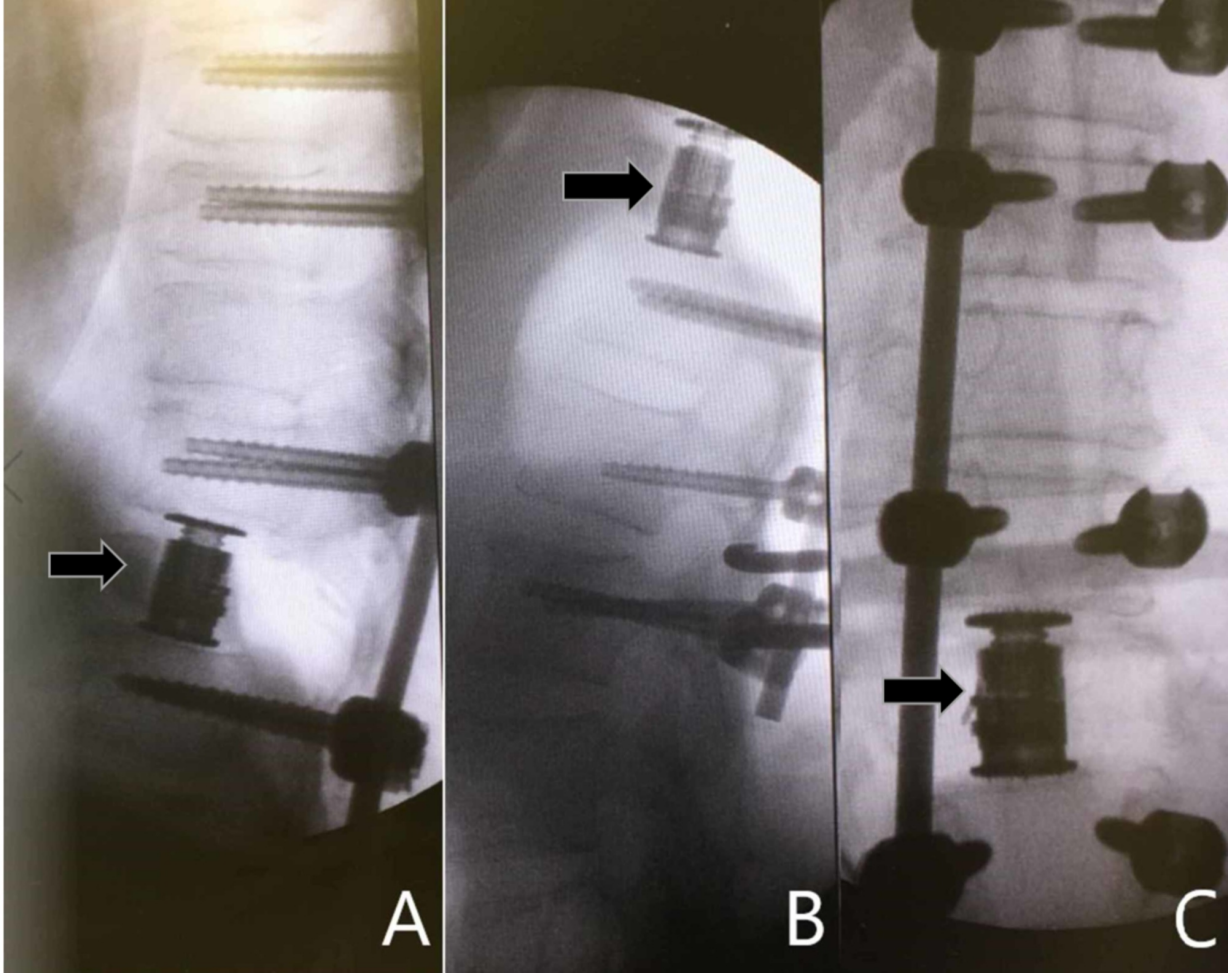
Intra-operative fluoroscopic lateral (A and B) and AP (C) X-ray images demonstrate pedicle screw placement bilaterally at T7, T8, T10, T12, L1, and L2 with an expandable interbody cage placement at T11 (black arrow). AP, anteroposterior

The patient was taken for an urgent posterior decompressive laminectomy from T9 to T11 , T9 left-sided pediculectomy with resection of intradural tumor (Figure 3), T11 right pediculectomy for a T11 corpectomy with anterior cage reconstruction, and instrumented pedicle screw fixation from T7 to L2 (Figure 4). The pathology from the T9 lesion demonstrated findings consistent with a meningioma while the T11 lesion confirmed metastatic non-small cell lung adenocarcinoma. The patient improved neurologically postoperatively and regained the ability to ambulate within one week of surgery.

## Discussion

Pathologically distinct spinal lesions in close anatomic proximity, but in two separate compartments are exceptionally rare in the absence of neurofibromatosis or von-Hippel-Lindau disease. During our literature review, the majority of concurrent spine tumors at either the same or adjacent spine levels involved meningiomas, schwannomas, and one case of a neurofibroma [1-5]. To our knowledge, we present the first case of concurrent metastatic lesion in addition to a meningioma involving the same area of the spine.

The MRI is widely considered to be the best preoperative imaging technique to diagnose spinal tumors. Deciphering spinal meningiomas from schwannomas on an MRI may be difficult when encountering an intradural, extramedullary mass. According to De Verdelhan et al.’s single institution review of 52 spinal meningiomas and schwannomas, meningiomas are more likely to occur within the thoracic spine, whereas schwannomas frequently arise within the cervical or lumbar spine. With a gadolinium contrast enhanced MRI, meningiomas tend to have homogenous, moderate enhancement with a dural tail [6]. Schwannomas typically have heterogeneous and significant enhancement on MRI with possible dumb-bell shape and foramen enlargement [7]. In terms of metastatic lesions to the spine, osseous metastases are the most common [8]. In cases of multiple spinal tumors, MRI is invaluable for preoperative planning. An MRI may be difficult to interpret in patients with concurrent tumors at the same level, and localized surgical exploration of both the intradural and epidural spaces may be prudent [9]. As Weil et al. demonstrated in 1990, two unexpected distinct etiologies may be discovered with a more thorough intradural and extradural exploration during surgery [9].

Our patient’s rapid neurologic decline likely stemmed from pre-existing severe cord compression from the presumably long-standing meningioma at T9 in addition to the metastatic lesion at T11. Concurrent tumors at nearby spinal levels present unique treatment challenges. Given the severe spinal stenosis present from both the intradural lesion at T9 and the anterior compressive lesion at T11 on preoperative MRI, we advocated for treatment of both disease foci with one operation. We performed a simultaneous posterior approach with a left pediculectomy for resection of the T9 meningioma and a right trans-pedicular T11 corpectomy for the metastatic lesion involving the anterior vertebral body. Pedicle screw fixation was performed from T7 to L2 given the potential instability caused by resection of the left pedicle at T9 and the right pedicle at T11 which were required for an adequate operative access window to simultaneously treat both pathologies.

## Conclusions

To our knowledge, this is the first documented case of a concurrent spinal meningioma and metastatic lesion. Different pathologies in nearby compartments present unique treatment challenges and require a thorough review of preoperative radiographic imaging. Given the presence of two separate lesions at nonconsecutive vertebral levels, a unique operative plan was formulated in order to treat both levels of critical spinal stenosis as well as the underlying pathology. 
